# The Effectiveness of Amitriptyline and Gabapentin in Treating Pomeranians with Chiari-like Malformation and/or Syringomyelia

**DOI:** 10.3390/ani15070992

**Published:** 2025-03-29

**Authors:** Ramona ter Maat, Kathelijn van Heusden, Larissa Hoogervorst-Spek, Marta Płonek, Paul J. J. Mandigers

**Affiliations:** 1 Department of Clinical Sciences, Faculty of Veterinary Medicine, Utrecht University, Yalelaan 108, 3584 CM Utrecht, The Netherlands; 2 Neurology Service, Evidensia Referral Hospital Arnhem, Meander 10, 6825 MB Arnhem, The Netherlands

**Keywords:** CBD oil, NSAID, opioids, furosemide, neuropathic pain

## Abstract

Background: Chiari-like malformation (CM) and syringomyelia (SM) are commonly observed disorders in the Pomeranians. Both disorders can lead to pain and diminish quality of life to varying extents. In veterinary medicine, affected dogs are often treated with gabapentin or pregabalin, which contrasts with human medicine, where the tricyclic antidepressants amitriptyline and nortriptyline are the first-choice treatments. Methods: affected dogs were treated with either amitriptyline or gabapentin. If a pre-syrinx was present, furosemide was also added. Owners were asked to score the clinical signs they observed (ORCS). Results: 90 Pomeranians were included, of which 62 received amitriptyline and 28 received gabapentin. Adding furosemide did not have an influence on the outcome. A logistic regression analysis with the number of ORCS, duration of ORCS, treatment (amitriptyline or gabapentin), and outcome (responder or non-responder) as the dependent variable revealed that the number of ORCS had an Odds of 1123 (*p* = 0.03). Duration of the ORCS (Odds 1.027; *p* = 0.45) and treatment group had a low influence (Odds 1.258; *p* = 0.65). Conclusions: In this study, amitriptyline demonstrated a moderate, though not statistically significant, more favorable effect on CM/SM pain compared to gabapentin. Adding furosemide to either of these two treatments did not influence the outcome. The outcome for dogs affected by CM/SM is not influenced by MRI grading; however, the number of ORCS and a longer duration negatively impact the outcome. A dose of 1 to 2 mg/kg body weight of amitriptyline administered twice daily proved to be more effective in several dogs.

## Simple Summary

Chiari-like malformation (CM) and syringomyelia (SM) are commonly observed disorders in the Pomeranians. Not all affected dogs develop clinical signs, but if they do, it can compromise the quality of life from very mild to severe. Affected dogs are often treated with a variety of medications, of which gabapentin and pregabalin are the most frequently used. This contrasts with humans, where amitriptyline and nortriptyline, both tricyclic antidepressants, are the first choice. To investigate the efficacy of amitriptyline and gabapentin, a group of 90 Pomeranians affected with CM and/or SM were treated with either one of these medications. In this study, amitriptyline showed a moderately more favorable effect on CM/SM pain than gabapentin. Dogs that showed frequent clinical signs for a short duration had a better outcome compared to dogs that exhibited more prolonged clinical signs.

## 1. Introduction

Chiari-like malformation (CM, also called canine Chiari or Chiari malformation) and syringomyelia (SM) are frequently seen disorders in the Pomeranian [1,2], Cavalier King Charles Spaniel (CKCS) [3,4,5,6], and several other small-sized dog breeds such as the Chihuahua, French Bulldog, and Griffon [7]. CM is characterized by a reduced volume of the caudal fossa and the caudal displacement of the caudal cerebellar vermis into or through the foramen magnum [8,9], along with an increased cerebellar volume [10]. Syringomyelia is defined as the presence of a fluid-filled cavity in the spinal cord (syrinx) and is believed to result from abnormal CSF flow, which may be related to hindbrain issues (CM) and/or the influence of the systolic and diastolic arterial pulse, as well as CSF pulse in the subarachnoid space [8,9,10,11,12,13]. One possible cause could be brachycephalism [14,15,16,17], but a recent study demonstrated that other factors also play a role [7]. The authors of the study observed syringomyelia in a dolichocephalic breed (miniature Dachshund) and two mesocephalic breeds (the Maltese dog and Yorkshire terrier) [7].

Not all dogs affected with CM and/or SM develop clinical signs. Exact figures are difficult to estimate. Clinical signs associated with CM are referred to as CM-associated pain (CM-P) [18] and can include vocalization during rapid postural changes as well as scratching or rubbing of the back of the head, ears, or face [18]. In the CKCS, the clinical signs caused by SM are allodynia, phantom scratching, cervico-torticollis, and thoracic limb and spinal weakness [18]. However, a recent study by Santifort et al. (2023) investigating the clinical signs in Pomeranians affected by CM and/or SM found that the most frequently reported owner clinical signs (ORCS) included scratching upon skin contact, rubbing of the head or ears, air licking, spontaneous vocalization, persistent licking of both front and/or hind paws, and phantom scratching [1]. In this study, phantom scratching, vocalization, head shaking, spontaneous signs of pain, and air licking were associated with SM. In the CKCS, there is a relationship between syrinx size (>4 mm) and ORCS [18]. In the Pomeranian, Santifort et al. (2023) did not find a statistically significant association between quantitative syrinx measurements and ORCS [1].

Effective management is essential to alleviate the clinical signs and improve the quality of life in affected dogs. Both medical and surgical options are available. In human medicine, surgery is the primary approach [19]. Although surgical treatment has been performed in veterinary medicine [20,21,22], it is not the primary approach. In dogs, the long-term outcome is not always favorable due to scar tissue formation at the surgical site [20,22]. In addition, and in contrast to humans, where syrinx resolution is common [23,24], this is infrequently the case for dogs. Furthermore, it must be emphasized that both CM and SM are frequently diagnosed disorders in our small-sized dog breeds [1,7,25,26]. For instance, the prevalence of CM in the CKCS is up to 99% and SM up to 46% [5,6]. In the Pomeranian, it was found to be 54.9% for CM and 23.9% for SM [1]. In humans, the prevalence is estimated to range from 0.24% to 3.6% [27].

The veterinary profession lacks both time and specialized staff to manage all affected cases. Furthermore, in veterinary medicine, we face considerable financial constraints associated with the diagnosis and management of disorders in dogs, which are, in most cases, absent in human medicine. Logically, veterinary medicine has focused on the medical treatment of CM and SM. Several studies have been published discussing the medical treatment of CM and/or SM [28,29,30,31,32,33], which has resulted in a treatment algorithm that can be accessed online (https://caninechiari.com/treatmentAlgorithm, accessed on 1 July 2024). Medical treatment can focus on managing neuropathic pain, decreasing cerebrospinal fluid (CSF) production, or both [31]. However, it should be noted that despite the high prevalence of CM/SM in dogs, the number of studies investigating the efficacy of the medications discussed, to the authors’ knowledge, is scarce.

Non-steroidal anti-inflammatory drugs (NSAIDs) are among the most prescribed drugs [29] but are often insufficient as monotherapy for neuropathic pain [31,34]. The most common medications for neuropathic pain in veterinary medicine are gabapentin and pregabalin [30,31,33]. This contrasts with human medicine, where the tricyclic antidepressants (TCAs) amitriptyline and nortriptyline are regarded as first-line therapies alongside gabapentin and pregabalin [35,36,37]. Other neuropathic treatments, such as topiramate, amantadine, opioids, cannabinoids, and serotonin-noradrenaline reuptake inhibitors, are often used in combination with other medications, yielding variable and sometimes poor results [30,31,34].

Amitriptyline and nortriptyline (TCAs) are not commonly prescribed in veterinary medicine. Amitriptyline antagonizes voltage-gated sodium channels, glutamate receptors, and NMDA receptors. Its mechanism of action is not completely understood, but its main effect is proposed to work by inhibiting the presynaptic reuptake of serotonin and noradrenaline, which are the neurotransmitters involved in the transmission of pain [30,31,38]. Nortriptyline is a metabolite of amitriptyline that inhibits noradrenaline reuptake, and it is also available as a medication itself [39]. TCAs are effective for achieving at least moderate pain relief in one out of every two to three human patients with peripheral pain and work better than gabapentin [35,39]. Aside from one study and some anecdotal reports, amitriptyline has not been adequately researched in dogs suffering from neuropathic pain due to CM/SM [30,31,40].

Reducing the production of cerebrospinal fluid (CSF) may help halt the progression of the syrinx and/or decrease its size. Several medications are available to reduce CSF production. A frequently used medication is the proton pump inhibitor omeprazole. Omeprazole reduces CSF production after intravenous administration [41], but it is questioned whether chronic oral administration has the same effect [42]. Furthermore, it should be noted that over-the-counter formulations of omeprazole contain magnesium, which reduces the uptake of gabapentin [31,43]. Other medications used to reduce CSF production are the diuretic furosemide [44,45] and the carbonic anhydrase inhibitor acetazolamide [45]. However, to date, there are no standardized studies, to the authors’ knowledge, that have demonstrated the effectiveness of these medications in dogs affected by SM.

The last group of medications is corticosteroids, often used as a last resort owing to their serious side effects. They can reduce neuropathic pain by preventing the release of proinflammatory mediators, decreasing substance P [46], and reducing CSF production [47].

Earlier, we described the phenotypic characteristics of CM and/or SM in the Pomeranians [1]. The aim of this study is to evaluate the therapeutic efficacy of amitriptyline and gabapentin, with or without furosemide, on ORCS in Pomeranians diagnosed with CM and/or SM.

## 2. Materials and Methods

### 2.1. Inclusion Criteria

All included Pomeranians were seen between 2015 and 2023 for CM/SM screening as described earlier [5]. Dogs could only be included if they (1) had ORCS associated with CM/SM [1] and (2) the MRI revealed either CM, SM, or a combination of both. Before inclusion, owners were asked to agree to participate in this study, and the owners’ informed consent was obtained. An approval from the animal welfare body of Utrecht University was requested but waived as the study adhered to the requirements on compliance with good laboratory practice for veterinary medicinal products set out in Annex II of Regulation (EU) 2023/183 of 23 November 2022 amending Regulation (EU) 2019/6 of the European Parliament and of the Council.

### 2.2. Data Set

Although ORCS were obtained at each consultation, all participating owners were asked to complete a standardized online questionnaire created with Qualtrics^®^ survey software (https://www.qualtrics.com/, accessed on 1 March 2024). The questionnaire was adapted from a previous study investigating the phenotypic characteristics of Pomeranians with CM and/or SM [1]. An example of the questionnaire is available in the Supplementary Materials Appendix SA. The answers were used to create a data set that included status (alive, yes/no), sex and neuter status, age at intake, age at follow-up, body weight, ORCS prior to treatment and ORCS after treatment, prescribed medication, and duration of treatment. For the ORCS, owners were also asked to score the duration of the ORCS in hours per day prior to and after treatment. Only the owners who completed this questionnaire were included in the study. The minimum treatment period was at least six months. Intake, MRI performance, medication prescription, and follow-up for all dogs were conducted by the last two authors (MP, PM).

### 2.3. MRI Studies

MRI studies were performed under general anesthesia (individualized anesthetic protocols) with a high-field MRI scanner (Canon Vantage Elan 1.5T MRI, Otawara-shi, Japan) at either the IVC Evidensia Referral Hospital Arnhem, The Netherlands, or the Department of Clinical Sciences, Utrecht University, The Netherlands (Philips Ingenia 1.5T MRI, Eindhoven, The Netherlands). Sequences obtained included a minimum of sagittal T2-weighted (T2W), sagittal T1-weighted (T1W), and transverse T2W or T1W sequences of the craniocervicothoracic region as described earlier [5]. For grading, we used the following classification as published previously [1]:

Chiari malformation

- CM 0: no cerebellar herniation or impaction.
- CM 1: cerebellar impaction.
- CM 2: cerebellar herniation.

Syringomyelia

- SM 0: no syringomyelia.
- SM 1: symmetric syrinx (i.e., circular, round).
- SM 2: asymmetric syrinx (e.g., extending into dorsal horn).

Additionally, the presence of a pre-syrinx was scored. A pre-syrinx is defined as a hyperintensity visible in the spinal cord on a T2W image, either in a sagittal or transverse view, and is believed to indicate interstitial, spinal cord oedema [48].

### 2.4. Treatment

After the first consultation, all owners were advised to give their dog either amitriptyline (Amitriptyline hcl cf tablet 10 mg, Centrafarm BV or Brocacef BV, The Netherlands) (starting dose 1 mg/kg body weight twice daily) or gabapentin (Gabapentin 50 mg/ml Focus Care Pharmaceuticals, The Netherlands or Gabapentin 20 mg/mL Pharmacy, Faculty of Veterinary Medicine, Utrecht University, The Netherlands) (starting dose 10 mg/kg bodyweight twice daily). Owners were free to choose the type of neuropathic pain medication. They were informed that both medications were effective, but the pharmaceutical formulation consisted of either a tablet (amitriptyline) or a liquid (gabapentin).

If a pre-syrinx was seen on the T2W sagittal or transverse image, furosemide (1 mg/kg body weight, twice daily) was added to the chosen treatment medication. Owners were informed that it could take two to three weeks for the treatment to become effective. If a dog receiving amitriptyline did not respond adequately, the owner was instructed to double the amitriptyline dose. Dogs on either amitriptyline or gabapentin, with or without furosemide, that the owner observed responding to the medication were classified as ‘responders’. Owners could rate the improvement on a scale from 0 (no effect) to 100 (complete effect). If an improvement of 50% or more was observed, the dog was classified as a responder. If an owner did not observe any response to the treatment, switched to another medication, or chose euthanasia, the dog was labeled a non-responder. If an owner chose a different medication, available alternatives included gabapentin, amitriptyline, an NSAID, and cannabidiol oil. All these dogs were still classified as non-responders.

### 2.5. Exclusion Criteria

Dogs diagnosed with intracranial space-occupying lesions or space-occupying lesions in the vertebral canal were excluded from the study. In addition, it was also excluded if essential information such as the presence and duration of ORCS, medication, exact dose, and effect were missing from the dog’s database.

### 2.6. Data Management and Statistical Analysis

All responses were automatically transferred to Microsoft^®^ Excel Redmond, WA, USA, where they were manually checked for duplicates. If a duplicate exists, only the most complete questionnaire is retained. The reliability of the results is manually checked and verified for compliance with the inclusion and exclusion criteria. The remaining data were analyzed with RStudio^®^ Boston, MA, USA. Descriptive statistics are used for CM/SM and sex. A Welch’s Two-Sample t-test is used for age as it is normally distributed, and a Mann–Whitney U-test is used for weight as it is not normally distributed. A Kruskal–Wallis test was used for the possible correlation between the grading of CM/SM and the response to treatment. The outcomes of dogs treated with amitriptyline, with or without furosemide, and gabapentin, with or without furosemide, were compared for these two groups separately using a Kruskal–Wallis test. If there is no difference between these groups, all amitriptyline-treated dogs (with or without furosemide) were compared to gabapentin-treated dogs (with or without furosemide) using logistic regression analysis. The analysis considered the number of ORCS, duration of ORCS, treatment (amitriptyline or gabapentin), and outcome (responder or non-responder) as dependent variables. Results are deemed statistically significant if *p* < 0.05.

## 3. Results

### 3.1. Study Population

A total of 290 owner-filled questionnaires were assessed. After removing the duplicate and incomplete responses, 269 remained. Of these 269 responses, 81 Pomeranians were excluded as they did not have CM/SM. Additionally, 4 Pomeranians with CM/SM were excluded because they had other neurological conditions, including a brainstem tumour (*n* = 1), cervical disc protrusions (*n* = 2), or meningoencephalitis of unknown origin (*n* = 1), that could have influenced the study outcomes. One Pomeranian that only presented with epilepsy as a symptom was also excluded. This resulted in 183 responses, of which 108 dogs received medical treatment. From this group, 18 dogs received an NSAID (*n* = 9), furosemide only (*n* = 5), or a combination of several other medications (*n* = 4), excluding them from this study.

Consequently, 90 dogs were available for analysis; 88 (98%) were purebred Pomeranians, one was a Pomeranian cross, and one was a Small German Spitz. The group consisted of 55 males and 35 females. The mean ± SD age of the dogs was 71 ± 29.5 months. The median body weight of the Pomeranians with CM/SM was 3 ± 1.6 kg.

### 3.2. MRI Grading

Table 1 shows the classification (CM, SM, or CM/SM) and severity of CM/SM (grade 1 or 2) within the total study population.

The T2W image revealed a pre-syrinx in 40 dogs. No relation was observed between pre-syrinx and SM grading.

### 3.3. ORCS

An overview of the most common ORCS is shown in Table 2.

### 3.4. Treatment Groups

Sixty-two dogs received amitriptyline (28 combined with furosemide), while 28 received gabapentin (12 combined with furosemide). Of the dogs receiving amitriptyline (with or without furosemide), six were classified as CM0, 49 as CM1, and seven as CM2. Of the dogs receiving gabapentin (with or without furosemide), three were classified as CM0, 18 as CM1, and seven as CM2. Of the dogs receiving amitriptyline (with or without furosemide), 18 were classified as SM0, 13 as SM1, and six as SM2. Of the dogs receiving gabapentin (with or without furosemide), nine were classified as SM0, six as SM1, and 14 as SM2. There was no statistically significant difference in the dogs’ grading or allocation to a treatment group (*p* = 0.295).

### 3.5. Effect of Age, Weight, and Grading on Response to Treatment

There was no statistically significant effect of age (*p* = 0.52) or weight (*p* = 0.39) on the response to treatment. Interestingly, there was no statistically significant difference in grading and response to treatment (*p* = 0.09). Of the nine dogs without CM, two were classified as SM1 and four as SM2. Three did not respond. In the larger group of 67 dogs classified as CM1, twelve SM0 dogs responded, while eight did not. All dogs classified as SM1 (*n* = 6) and SM2 (*n* = 27) responded to the treatment. All four dogs classified as CM2, which included two SM0 and two SM2, also responded to treatment.

### 3.6. Effect of Number of ORCS, Duration of ORCS, and Response to Treatment

A statistically significant difference was observed between the number of reported ORCS and response to treatment (*p* = 0.003), as well as the duration of the ORCS (*p* = 0.035). This indicated that the more ORCs a dog possessed or the longer they persisted throughout the day, the less likely the dog was to respond to treatment. The mean (±SD) number of ORCS for responders was 7.5 ± 4.8 compared to 10.2 ± 4.3 for the non-responders. The mean (±SD) duration of ORCS was 5 ± 6.8 for the responders and 10.1 ± 7.5 for the non-responders. Again, there was no relation between grading severity and the number (*p* = 0.825) and duration (*p* = 0.328) of ORCS, respectively.

### 3.7. Response to Treatment

Sixty-two dogs received amitriptyline, of which twenty-eight also received furosemide. Twenty-eight dogs received gabapentin, with twelve also receiving furosemide. There was no statistical difference in the treatment response for dogs receiving only amitriptyline and those receiving a combination with furosemide (see Table 3). A similar observation was noted for dogs treated with gabapentin, whether or not it was combined with furosemide.

Since adding furosemide had no effect, all dogs treated with amitriptyline were compared to those treated with gabapentin. A logistic regression analysis with the number of ORCS, duration of ORCS, treatment (amitriptyline or gabapentin), and outcome (responder or non-responder) as the dependent variable revealed that the number of ORCS had an Odds of 1123 (*p* = 0.03). Duration of the ORCS (Odds 1.027; *p* = 0.45) and treatment group had a low influence (Odds 1.258; *p* = 0.65).

The dosage of amitriptyline was known for 45 of the 62 dogs treated with amitriptyline. There was a statistically significant difference between the two groups (1 mg/kg body weight versus 2 mg/kg body weight). Seven of the twenty-eight dogs receiving 1 mg/kg body weight did not respond compared to one non-responder of the thirteen dogs that received 2 mg/kg body weight (*p* = 0.016). If dose was added to the above-described logistic regression analysis, the odds that a dog responded to treatment with a dose of 2 mg/kg bodyweight increased (*p* = 0.06).

Fifteen of the 35 non-responding dogs were euthanized. The mean (±SD) number of ORCS was 12.9 ± 3.9, and the mean (±SD) duration was 15.2 ± 6.7 h per day. All 15 dogs received several other medications, such as CBD oil, opioids, NSAIDs, and corticosteroids, before euthanasia was elected. Unfortunately, the exact dose of the medication was unknown for all dogs.

Five of the dogs that did not respond were switched to either gabapentin or amitriptyline treatment, and all five responded moderately. Non-responding dogs were generally treated with a variety of additional medications and products, such as CBD oil, opioids, NSAIDs, and corticosteroids.

### 3.8. Reported Side-Effects

A small number of owners reported side effects. Drowsiness, lethargy, and nausea were reported in seven dogs treated with gabapentin. Lethargy, nausea, and aggression were noted in three dogs treated with amitriptyline. The dose for all three dogs was 1 mg/kg of body weight, administered twice daily. Polyuria/polydipsia was observed in two of the 40 dogs treated with furosemide. There are two gabapentin formulations available. One is registered for humans (Gabapentin 50 mg/mL Focus Care Pharmaceuticals) with the taste and smell of oranges, and the other is a tasteless version (20 mg/mL) made by the pharmacy of the Faculty of Veterinary Medicine, Utrecht University. The latter appeared to be more palatable.

## 4. Discussion

Both CM and SM can cause clinical signs. In this study, 90 Pomeranians with clinical signs were treated with either amitriptyline or gabapentin. This is the first larger study investigating the use of amitriptyline in dogs. As a study setup, a prospective positive controlled study was chosen in which owners were free to choose the medication. Most owners chose amitriptyline. A likely reason is the ease of administration, as amitriptyline is available in a 10 mg tablet. Most Pomeranians weigh 2.5 to 3 kg, and most dogs received a quarter tablet twice daily (if the starting dose was 1 mg/kg bodyweight twice daily). Gabapentin is available in 100 mg capsules, but logically, this would not be an option as the exact dosage is impossible. For this reason, the liquid variant was chosen, but not all dogs appreciated the fruit taste of the human-registered version. Although a veterinary alternative without taste was available, the cost of this formulation was considerably higher. We added furosemide to the chosen medication if a pre-syrinx was visible on T2W images of the spinal cord. Furosemide can decrease CSF production [44,45], but again, there are no standardized studies that demonstrate the effectiveness of this medication in dogs affected by SM. The analysis of the 40 dogs treated with amitriptyline/gabapentin and furosemide did not reveal a statistically significant response in either group. It should be noted that during the analysis of this study, follow-up MRI results were not available. It is possible that owners may not observe a clinical response, but MRI changes that could benefit the dog’s long-term outcome might exist when furosemide is added to the treatment. In this study, the number of dogs that responded favorably in the amitriptyline group, although not statistically significant, was slightly higher compared to the gabapentin group. In a small number of dogs, the effect was even higher if the amitriptyline dose was doubled from 1 mg/kg bodyweight twice daily to 2 mg/kg bodyweight twice daily. The dose of 1 mg/kg was chosen based on two reports describing the use of amitriptyline in dogs. Both studies used a dose near 1.3 to 1.5 mg/kg bodyweight twice daily [40,49]. Based on this study, it seems safe to conclude that if a dose of 1 mg/kg body weight twice daily does not work, it is wise to double the dose before deeming the drug ineffective.

In the logistic regression model, it became clear that both the number of ORCS and the duration of ORCS are important considerations. Dogs that exhibit a high number of ORCS during the day can be most effectively treated with higher doses of amitriptyline or a more tailored combined treatment. For clinicians, it is also vital to recognize that Pomeranians with a lower number and shorter duration of ORCS tend to have better outcomes when treated. Fifteen dogs were euthanized as they did not respond to the given treatment. These 15 dogs had a high number of ORCS of a lengthy duration. Although all 15 dogs received several other medications, it is impossible to differentiate them further from the responding dogs. It is possible that frequent lengthy ORCS forced the owners to elect for euthanasia of their dog.

Interestingly, as in the previous study of Santifort et al., 2023 [1], MRI grading did not predict which ORCS would be visible and what the clinical outcome of the patient would be. This is in contrast with the CKCS, in which a relation between syrinx size (>4 mm) and ORCS exists [18]. This seems to suggest that the etiology and pathogenesis are different for these two breeds. Whether amitriptyline provides similar results in the CKCS cannot be concluded based on the findings of this study.

Similar to the study by Santifort et al., 2023 [1], the most frequently observed ORCS in this study were: (1) scratching on skin contact, rubbing of the head, (2) air licking, (3) persistent licking of the front and hind paws. In this study, up to 59% of the dogs showed excessive swallowing, yawning, panting, and protrusion of the tongue. This is most likely attributable to a small adjustment made in the questionnaire used. By explicitly adding options such as ‘panting’ and ‘yawning’, owners may have been more inclined to select these signs than when they need to provide these themselves.

This study has some limitations. First, it is possible that some degree of subjectivity from the owners exists. Using more objective tools to analyze the responses would enhance objectivity. Second, several dogs could not be included in the analysis because the owners deviated from treatment protocols.

## 5. Conclusions

In this study, amitriptyline showed a moderate, though not statistically significant, more favorable effect on CM/SM pain compared to gabapentin. Adding furosemide to either of these two treatments did not influence the outcome. The prognosis for dogs affected by CM/SM is not influenced by MRI grading, but the number of ORCS and a longer duration do have a negative effect on the outcome. A dose of 1 to 2 mg/kg body weight of amitriptyline twice daily was effective in several dogs.

## Figures and Tables

**Table 1:** MRI grading of the 90 included Pomeranians according to Santifort et al., 2023 [1].

Grading	SM 0	SM 1	SM 2	Total
CM 0	0 (0%)	3 (3.3%)	6 (6.7%)	9 (10.0%)
CM 1	20 (22.2%)	12 (13.3%)	35 (39%)	67 (74.5%)
CM 2	7 (7.8%)	1 (1%)	6 (6.7%)	14 (15.6%)
Total	27 (30.0%)	16 (17.6%)	47 (52.4%)	90 (100%)

**Table 2:** Summary of the most common reported ORCS in the affected dogs.

ORCS	Number & Percentage	Explanation
Scratching with skin contact, rubbing of the head	83 (60.6%)	Scratching of the neck or shoulder region with direct skin contact; rubbing the nose, mouth, or ears against surfaces, like floors or walls.
Excessive swallowing, yawning, panting, protrusion of the tongue	81 (59.1%)	Excessive swallowing not related to food or water intake; excessive yawning; excessive panting unrelated to heat or exercise; repeated protrusion of the tongue or licking around the mouth.
Air licking	74 (54.0%)	Frequent and repetitive licking of the air.
Persistent licking of front and hind paws	71 (51.8%)	Persistent licking or gnawing of the front or hind paws.
Lethargy	70 (51.1%)	Withdrawal behavior, including hiding behind or underneath furniture; at times very timid.
Head shaking	62 (45.3%)	Spontaneous, recurrent shaking of the head.
Disturbed sleep	60 (43.8%)	Restless sleep; sleeping with an elevated head position.
Facial expressions suggestive of pain	55 (40.1%)	Excessive squinting or other facial indicators of discomfort or pain.
Phantom scratching	42 (30.7%)	Scratching motions directed toward the neck area without skin contact.
Pain responses to external stimuli	42 (30.7%)	Signs suggestive of pain in response to external stimuli, like physical touch.
Neck and/or back problems	41 (29.9%)	Signs of neck or back pain, limited movement, reluctance or inability to jump, a bent neck or back posture.
Spontaneous signs of pain	38 (27.7%)	Signs suggestive of pain independent of external stimuli, like physical touch.
Aggression	36 (26.3%)	Aggressive responses toward the owner, other animals, or other individuals, like housemates or visitors.
Fly-catching or tail-chasing behavior	30 (21.9%)	Recurrent episodes of biting in the air (mimicking fly-catching) or chasing the tail.
Weakness and/or impaired coordination	30 (21.9%)	Weakness of the front or hind limbs, inability to support bodyweight, limping, or signs of ataxia.
Hyperexcitability	29 (21.2%)	Heightened excitability, such as vocalisation (screaming) when excited or excessive activity.
Epilepsy	15 (10.9%)	Seizure episodes or epileptic events.
Paroxysmal dyskinesia	15 (10.9%)	Recurrent episodes of muscle cramping and dystonic movements.

**Table 3:** Response to treatment: There is no difference in response between dogs receiving furosemide and those not receiving it. A small number of dogs responded favorably to amitriptyline compared to gabapentin.

Medication	Responders	Non-Responders	Total
Amitriptyline	21 (62%)	13 (38%)	34
Amitriptyline + furosemide	19 (68%)	9 (32%)	28
Gabapentin	9 (56%)	7 (44%)	16
Gabapentin + furosemide	6 (50%)	6 (50%)	12
Total	55	35	90

## Data Availability

The data used for this study can be shared upon request.

## Supplementary Materials

The following supporting information can be downloaded at: https://www.mdpi.com/article/10.3390/ani15070992/s1, Appendix SA. Survey.

## Abbreviations

The following abbreviations are used in this manuscript:

CBD	Cannabidiol
CKCS	Cavalier King Charles Spaniel
CM	Chiari malformation
CSF	cerebrospinal fluid
ORCS	owner-reported clinical signs
NSAID	non-steroidal anti-inflammatory drugs
SD	standard deviation
SM	Syringomyelia
TCA	tricyclic antidepressant
THC	Tetrahydrocannabinol
