# Supplementary material for: The Effectiveness of Amitriptyline and Gabapentin in Treating Pomeranians with Chiari-like Malformation and/or Syringomyelia
Source: Animals (Basel). 2025 Mar 29;15(7):992. doi: 10.3390/ani15070992 (PMC11987954; doi:10.3390/ani15070992)
Supplement: Supplementary file 1 [file animals-15-00992-s001.zip › animals-3536815-supplementary.pdf]

Appendix SA

Survey (translated from Dutch)

Thank you very much for your participation. The purpose of this survey is to gain insight into the course over time and/or the effect of medication. It is possible that you will receive this request multiple times if you are known to us with several dogs. Unfortunately, it is also possible that your dog already died. In that case, your participation is still very important and we ask you to complete the survey as best you can. It is important that you enter the unique code given to you. If you have several dogs, you do not have to enter your dog's details again each time. The first question we ask is whether you agree to this research. If you do not agree, the survey will close. If you agree, the survey will start, but rest assured: we will never disclose any data about you or your dog. All data will be treated confidentially, and all privacy sensitive data will never be shared with third parties. Completing the survey will take approximately 10 minutes. Thank you very much for your participation.

Q0 Do you agree to participate and that we use the information submitted for research?

No --> end of survey

Yes --> Q1

Q1 Please enter the unique code you received from us

Q2 What is your dog's name?

Q3 Just to be sure: what is your surname?

Q3-1 Is your dog still alive? Y/N

    If N

    Q3-2 When did your dog die?

    Q3-3 What did he/she die from / due?

    Q3-4 What was your dogs bodyweight at that time?

    if Y --> Q3-5

Q3-5 What is your dogs current bodyweight?

Q5 Did your dog suffer from clinical signs suggestive for CM/SM (as informed). Please see also information sheet... Y/N

    if N --> Q6

Q5-1 Please mark the clinical signs applicable. You can mark several.

▢ Expressions of pain when touched

- ▢ Shaking the head
- ▢ Frequently rubbing the ears on the ground
- ▢ Frequently rubbing the mouth or nose on the ground
- ▢ Frequently tongue-licking
- ▢ Licking the air
- ▢ Frequent swallowing
- ▢ Scratching the shoulder/neck
- ▢ Scratching in the air (so the paw does not touch the skin)
- ▢ Spontaneous expressions of pain without the dog being touched
- ▢ Expressions of neck pain (difficulty with/not wanting to bend the neck)
- ▢ Expressions of back pain (walking stiffly, not wanting to jump)
- ▢ Walking with a crooked/bent neck or back
- ▢ Aggression towards other dogs or other animals
- ▢ Aggression towards me as the owner
- ▢ Aggression towards my housemates (partner or children)
- ▢ Sleeping with my head held high/sleeping with my head on a raised surface
- ▢ Muscle weakness in the forehand (sinking through the forehand)
- ▢ Frequent licking/gnawing at the front gates
- ▢ Frequent licking/gnawing at the hind legs
- ▢ Frequent squinting with the eyes
- ▢ Lameness with one or more legs
- ▢ Frequent yawning
- ▢ Restless sleep
- ▢ Epilepsy
- ▢ Tail chasing
- ▢ Attacks of cramping
- ▢ Fly biting
- ▢ Screaming when excited
- ▢ Being very busy / acting busy
- ▢ Isolating oneself / lying under a cupboard, couch or bed
- ▢ Occasionally acting very quietly
- ▢ (Attack-like) walking unsteadily
- ▢ Muscle weakness in the hindquarters
- ▢ Excessive panting (independent of movement/heat)

▢ Other, please explain further _______

Q5-2 Please mark the three clincal signs you saw the most.

(same list Q5-1)

Q5-3 How many hours per day did you observe these clinical signs?

Q5-4 When we discussed the findings. Did you start a treatment? Y/N

if Y --> Q5-5

if N --> Q7

Q5-5 Which medication did you choose?

▢ Gabapentin: The dose was:

▢ Amitriptyline. The dose was:

▢ Did this include furosemide?

▢ I gave something else:

Q5-6 On a scale of 0 to 100. Did the medication work? 0 means not at all, 100 means perfectly.

Q5-7 What is the current situation?

o He responds well to the medication, and I am satisfied

o He did not respond well to the medication, and I had to supplement the medication

Q5-8 You indicated that he did not respond. What did you do?

o He did not respond at all and eventually I had to put him to sleep. Please provide more info if necessary. --> Q8-1

Q6 You indicated that your dog did not present with clinical signs suggestive for CM/SM. How is the current situation? --> Q6-1 and further

Q6-1 What kind of clinical signs do you see? --> same list as Q5-1

Q6-2 Please mark the three clinical signs you saw the most.

(same list Q5-1)

Q6-3 How many hours per day did you observe these clinical signs?

Q6-4 When we discussed the findings. Did you start a treatment? Y/N

Q6-5 Which medication did you choose?

▢ Gabapentin: The dose was:

▢ Amitriptyline. The dose was:

▢ Did this include furosemide?

▢ I gave something else:

Q6-6 On a scale of 0 to 100. Did the medication work? 0 means not at all, 100 means perfectly.

Q6-7 What is the current situation?

Q8-1 You indicated that your dog did not respond to treatment. Please mark the clinical signs you observed --> same list Q5-1.

Q8-2 Please mark the three clinical signs you saw the most.

(same list Q5-1)

Q8-3 How many hours per day did you observe these clinical signs?

Q8-4 When we discussed the findings. Did you start a treatment? Y/N

Q8-5 Which medication did you choose?

▢ Gabapentin: The dose was:

▢ Amitriptyline. The dose was:

▢ Did this include furosemide?

▢ I gave something else:

Q8-6 On a scale of 0 to 100. Did the medication work? 0 means not at all, 100 means perfectly.

Q8-7 What is the current situation?

Q7 You indicated that your dog did not develop clinical signs. This is of course great. If you have any remark / question, please raise it.

Q9 Do you have any additional remarks?
